# Genomic characterization of enteropathogenic *Escherichia coli* (EPEC) of avian origin and rabbit ileal loop response; a pet macaw (*Ara chloropterus*) as a possible zoonotic reservoir

**DOI:** 10.1080/01652176.2020.1845916

**Published:** 2020-12-03

**Authors:** André Becker Simões Saidenberg, Arnoud H.M. van Vliet, Paulo Eduardo Brandão, Lilian Rose Marques de Sá, Marcos Paulo Vieira Cunha, Roberto M. La Ragione, Terezinha Knöbl

**Affiliations:** a School of Veterinary Medicine and Animal Science, University of São Paulo, São Paulo, Brazil; b Department of Pathology and Infectious Diseases, School of Veterinary Medicine, Faculty of Health and Medical Sciences, University of Surrey, Guildford, Surrey, UK

**Keywords:** Macaw, *Ara chloropterus*, pittacine birds, typical EPEC, ST40 *Escherichia coli*, O109:H2, zoonosis, whole-genomic sequencing

## Abstract

Enteropathogenic *Escherichia coli* (EPEC) constitutes one of the main causes of mortality in children in low- to medium-income countries. Diverse animal species have been linked as reservoirs, including birds. The aim of this study was to describe the genomic and phylogenetic features of an EPEC recovered from a pet macaw and further characterizing the macro and microscopic lesion in a rabbit ileal loop experimental model. The isolate was whole-genome sequenced (WGS) obtaining its genotypic and phenotypic *in silico* characteristics and inoculated in a rabbit experimental model with subsequently evaluating the strain’s pathogenicity by scanning electron microscopy (SEM) and histopathology. The isolate was characterized as O109:H21-B1-ST40 typical EPEC, harboring several virulence factors of diarrheagenic *E. coli*. The macaw EPEC genome was located in a monophyletic clade of human and animal ST40 EPEC sequences. *In vivo* inoculation demonstrated severe hemorrhage with SEM and histopathological analysis confirming these lesions to be associated with intra-epithelial lymphocytes. Therefore, the isolate not only shared several genotypic and phylogenetic similarities with EPEC that affects humans and animals, but was able to induce severe tissue injury in a mammal model. These findings highlight the underrated role of pet birds as zoonotic reservoirs and the diversity in virulence factors being unraveled by new WGS studies.

## 1. Introduction

Diarrheagenic *Escherichia coli* pathotypes are one of the leading causes of morbidity and mortality in humans, particularly in low- to medium-income countries, such as in Latin America. These pathotypes account for thousands of deaths in children under 5 years of age where it remains a continuous challenge to control due to social and economic issues connected to lack of education related to personal hygiene, inadequate sanitation, and poor quality of water (Lozer et al. 2013).

Enteropathogenic *E. coli* (EPEC) is one of the main diarrheagenic *E. coli* pathotypes causing attaching and effacing (A/E) lesions with the classical definition of the characteristic intimate adherence and effacement of the intestinal microvilli. Typical strains possess the virulence factors intimin (*eae* gene) and bundle-forming pili (*bfp* gene), while atypical ones lack the latter (Pearson et al. 2016). Several animal species (livestock, wild animals, and pets like dogs and cats) have been identified as potential reservoirs of EPEC, which are genetically similar to those isolates from human clinical cases, reinforcing the potential for zoonotic transmission and its importance in the ‘One Health’ initiative (La Ragione et al. 2002; Torres 2017).

Sanches et al. (2017) examined a total of 516 fecal samples isolated from captive birds belonging to 10 orders (including 70 species) and reported the presence of typical EPEC in Psittaciformes (14.4%) and Columbiformes (1.38%). Psittacine birds (macaw, parrot, and parakeet) are frequent household pets that closely interact with humans and have been well established as reservoirs for zoonoses such as psittacosis (Halsby et al. 2014). Molecular studies on diarrheagenic *E. coli* pathotypes have described severe cases of diarrhea and sepsis in parrots as well as a carrier state as zoonotic reservoirs (Saidenberg et al. 2012; Gioia Di-Chiacchio et al. 2018). However, there is a paucity of whole-genome sequencing (WGS) data on parrots derived EPEC strains. Therefore, a better understanding of the genomics of these strains, through more in-depth comparisons with human EPEC strain sequences, could help to elucidate the zoonotic potential. We performed WGS on a typical EPEC strain isolated from a pet macaw describing the genetic features of this isolate, comparing it with genome sequences from humans and other animals available in public databases, in addition to evaluating the virulence potential in a rabbit ileal loop surrogate model of infection.

## 2. Materials and methods

### 2.1. Sample collection and processing

An adult (unknown gender) red and green macaw (*Ara chloropterus*) was presented for a general health check-up at a private veterinary practice and a cloacal swab was obtained for routine microbiological culture. The bird was healthy (good body score and weight 1180 g – a normal value for an adult individual of the species) and alert, not showing any signs of enteric disease (well-formed feces). No other pets were present in the household and the bird had been acquired from a reputable breeder without any illness history.

The swab was cultured on BHI medium (brain and heart infusion, DIFCO-BBL, Detroit, MI, USA) at 37 °C, aerobically, 24 h and then streaked onto MacConkey agar plates (DIFCO-BBL) and incubated for 24 h at 37 °C, aerobically. *E. coli* was isolated in a pure culture and colonies were stored in Luria-Bertani medium with 30% glycerol and frozen at −80 °C and identified as FMVZ-USP MA-81.

### 2.2. Screening for the presence of diarrheagenic E. coli (DEC) genes

*E. coli* colonies were subjected to the PCR amplification for the detection of some DEC virulence genes, according to the method described by Costa et al. (2010). The DNA extraction was performed as described by Boom et al. (1990) and PCR for amplification included *eae* (454 bp), *bfp* (550 bp), *stx*1 (349 bp), and *stx*2 (110 bp) genes. *E. coli* K12 DH5α (Gibco BRL) and O137:H6 tEPEC/STEC (CA14) are employed as negative and positive control (Gioia Di-Chiacchio et al. 2018). The amplification mixture consisted of Tris-HCl buffer (pH 8.3) 10 mM, MgCl_2_ deoxynucleotide triphosphates 200 mM, pairs of primers, Taq DNA polymerase 0.5 U, and ultrapure water autoclaved in a final volume of 25 µL. Amplified products were separated in 1.5% agarose gel and examined after stained with BlueGreen^®^ (LGC Biotecnologia, Cotia, São Paulo, Brazil). A 100 bp DNA ladder (LGC Biotecnologia, Cotia, São Paulo, Brazil) was used as a molecular size marker.

### 2.3. Dna preparation and whole-genome sequencing

*E. coli* culture was plated onto LB agar (DIFCO-BBL) and incubated at 37 °C overnight, aerobically. A single colony (MA-81) from a pure culture was selected for genomic DNA extraction and purification using PureLink Genomic DNA purification Kit (Invitrogen, Carlsbad, California, USA) following the manufacturer's recommendations. Libraries were generated with a Nextera XT DNA Library kit (Illumina^®^, San Diego, CA, USA) according to the manufacturer instructions and paired-end (2 × 75 bp) sequenced in an Illumina MiSeq platform (Illumina, San Diego, CA, USA).

### 2.4. De novo assembly, annotation, and identification of genotypic and phenotypic genomic features

Quality control checks on raw sequence data were performed using FastQC (v.0.72). Read trimming was carried out using Trimmomatic (v.0.38.0). (Bolger et al. 2014). The genome was assembled *de novo* using Shovill software version 1.0.4 (https://github.com/tseemann/shovill), and annotated with PROKKA version 1.13 (Seemann 2014), followed by *in silico* identification of serotype, phylogroup, plasmid replicons, antimicrobial resistance and virulence factors using ABRicate version 0.9.0 (https://github.com/tseemann/abricate) with the VFDB, SerotypeFinder, EcOH, Plasmidfinder, Resfinder and NCBI Bacterial Antimicrobial Resistance Reference Gene Databases. Multi-locus sequence typing (MLST) was done using MLST version 2.16 (https://github.com/tseemann/mlst).

### 2.5. Rabbit ileal loop inoculation

To determine the isolate’s pathogenicity, an *in vivo* rabbit ileal loop inoculation model was utilized (protocol adapted from Trabulsi 1964), and small intestine samples examined using scanning electron microscopy (SEM) and histopathology. All studies were conducted under the jurisdiction of an animal license and abided by laboratory animal welfare standards of Brazil (ethical committee CEUA 7423290414 and 7843030717 – FMVZ- University of São Paulo).

Prior to the surgery, New Zealand white rabbits (males, 6–8 weeks of age, weighing 1.9–2.3 kg) were screened for absence of coccidia (parasitological examination) and *eae + E. coli* by PCR (Costa et al. 2010). Briefly, after 8 h of fasting, the rabbit was anesthetized with combination of acepromazine (0.1 mg/kg BW) (0.2% Acepran^®^ Univet S/A, São Paulo – SP, Brazil), fentanyl citrate (0.3 mL/kg BW) (Fentanyl^®^ 50 µG/mL, Janssen-Cilag Farmacêutica Ltda, São Paulo – SP, Brazil) and tiletamine/zolazepam (20 mg/kg BW) (50 Zoletil^®^, Virbac Brazil Industry and Commerce Ltd., São Paulo – SP, Brazil), via intramuscular route, followed by deep isoflurane inhalation anesthesia (1,5%) for the entire procedure. During a laparotomy, the distal portion of ileum was washed three times with sterile saline. Four segments of the ileum measuring 5 cm in length were ligated on both ends, with 3 cm apart inter-loops. Each loop was inoculated separately with 1 mL (at a concentration of 1 × 10^6^ CFU/mL in LB broth, cultivated by 16 h, aerobically, at 37 °C) of each suspension as follows: the MA-81 macaw strain, followed by ATCC *E. coli* K-12 DH5α (Gibco BRL, non-pathogenic strain; negative control) and E2348/69 (tEPEC strain; *eae*+, *bfp*+; positive control) (Levine et al. 1985). Sterile PBS was inoculated in the fourth loop. Sodium dipyrone (25 mg/kg BW) was used for post-surgical analgesia. After 12 h, the rabbit was humanely euthanized with an overdose of anesthetic (60% isoflurane and potassium chloride 20 mg/kg BW) (Brazil, Ministério da Ciência, Tecnologia e Inovação, 2018).

A *post-mortem* examination was then performed, gross observations recorded, and tissues harvested for histopathology and scanning electron microscopy.

### 2.6. Histopathological evaluation

Sections of the inoculated intestinal loops were collected in 10% neutral buffered formalin and included in paraffin after dehydration and diaphanization. The paraffin blocks were sectioned in Leica RM2145 microtome (Leica Biosystems, Nußloch, Germany) and stained with hematoxylin and eosin following standard procedures. Tissues were then examined using standard light microscopy (Eclipse NiU Nikon, with camera DS-U3, Software Ni Elements; Nikon Corporation, Tokyo, Japan).

### 2.7. Scanning electron microscopy (SEM)

At *post-mortem* examination, other sections of the inoculated intestinal loops were harvested and fixed in 2.5% glutaraldehyde (v/v) in 0.1 M phosphate buffer, washed with 0.1 M sodium cacodylate buffer, followed by 1% osmium tetroxide (OsO_4_) (v/v), and ethanol dehydrated solutions. Tissues were dried using the critical point method and mounted onto SEM stubs sputter coated with gold and using a Quanta 250 scanning electron microscope device (FEI Company, Hillsboro, Oregon, USA), at 12.5 kV and working distance of 7 mm.

### 2.8. Core and whole genome phylogenetic analyses

Complete and draft sequences were used for the comparative phylogenomics of 26 other *E. coli* genomes from Genbank/RefSeq/Enterobase downloaded as FASTA files (Supplementary Table 1). These included prototypic intestinal pathogenic *E. coli* human strains for general comparisons and sequences from livestock, wildlife, and human clinical cases selected by their similarity to the current study’s strain due to the presence of the same somatic (O) and/or flagellar (H) antigens and/or MLST group assignment.

**Table 1.** Assembly metrics and *in silico* features of the sequenced macaw isolate.

FMVZ-USP NL81 – EPEC macaw	GenBank accession number: SAMN11605913
*Assembly statistics*	
Contigs	285
Largest contig	265349
Total length	5061182
N50	106802
L50	15
GC (%)	50.35
# N's per 100 kbp	0
Gene	4812
CDS	4751
tRNA	57
rRNA	3
tmRNA	1
Repeat regions	2
Coverage	160x
*Serotype*	O109:H21
*MLST (Achtman scheme)*	40
*Phylogroup*	B1
*Resistance genes*	*mdf(A)*
*Plasmids*	IncFIB, IncFIC(FII), IncFII

To compare the core genome, single nucleotide polymorphisms (SNPs) analysis was performed with the ParSNP program (Treangen et al. 2014) and using the genome sequence of EPEC-E2348/69 from Genbank (NC_011601) as a reference. For the whole genome comparative analysis, an alignment-free method feature frequency profiling (FFP) was employed with the FFPry program (van Vliet and Kusters 2015). ITOL v.4 (https://itol.embl.de/) program was used for visualization and adding the available metadata for the ParSNP and FFPry trees. The sequencing reads and genome assembly of *E. coli* strain FMVZ-USP MA-81 have been uploaded in the SRA (SRR9045867) and NCBI (SAMN11605913) genome public databases.

## 3. Results

### 3.1. In silico genotyping

*In silico* WGS testing identified this strain as serotype *E. coli* O109:H21, phylogroup B1, MLST ST40 (Achtman scheme) (see Table 1 assembly metrics and *in silico* features) and also confirms that MA-81 was a typical EPEC by presence of *eae* and *bfp* genes.

Many genes associated with virulence factors of diarrheagenic *E. coli* were detected (see Supplementary Table 2), including genes involved in the processes of adherence, regulation, type 3 secretion system proteins, production of toxins and proteases and type three translocated proteins. WGS also revealed the presence of plasmids (IncFIB, IncFIC(FII), IncFII), and the gene *mdf*(A) conferring resistance to macrolide, lincosamide, and streptogramin B antibiotic classes (Table 1).

### 3.2. Macroscopic changes and SEM

**Figure 1.**
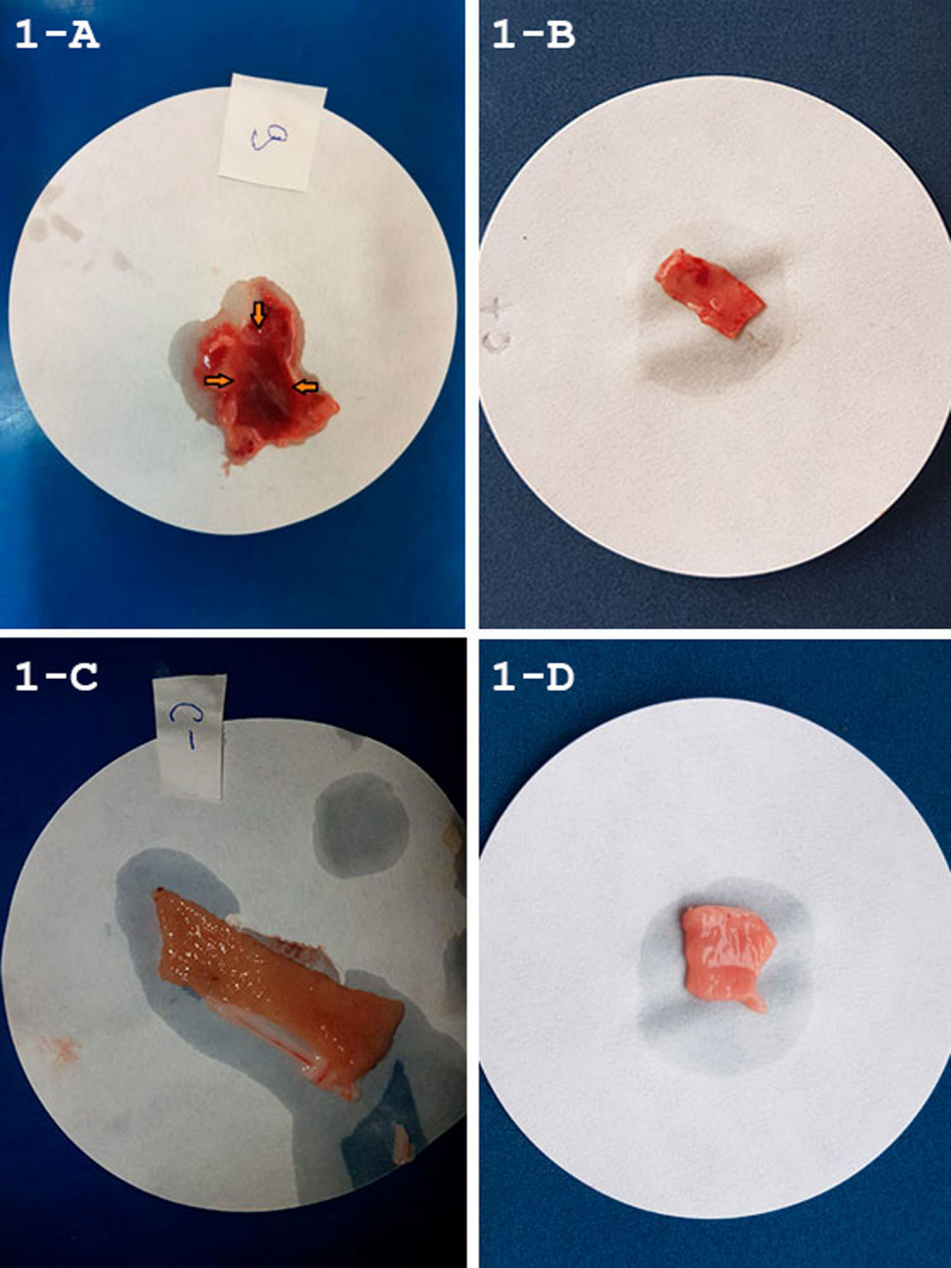
(A–D) Gross evaluation of inoculated rabbit ileal loops. Macroscopic observation of the effects of the ileal loops model 12 h. post-inoculation. MA-81 induced extensive fluid accumulation, hemorrhage with mucous and edema of the intestinal mucosa (**A** orange arrow). EC.2348/69 (positive control) produced slight amount of mucus and mucosa was hyperemic (B). Absence of macroscopic alterations in the negative controls K12-DH5α and PBS, respectively (C and D).

**Figure 2.**
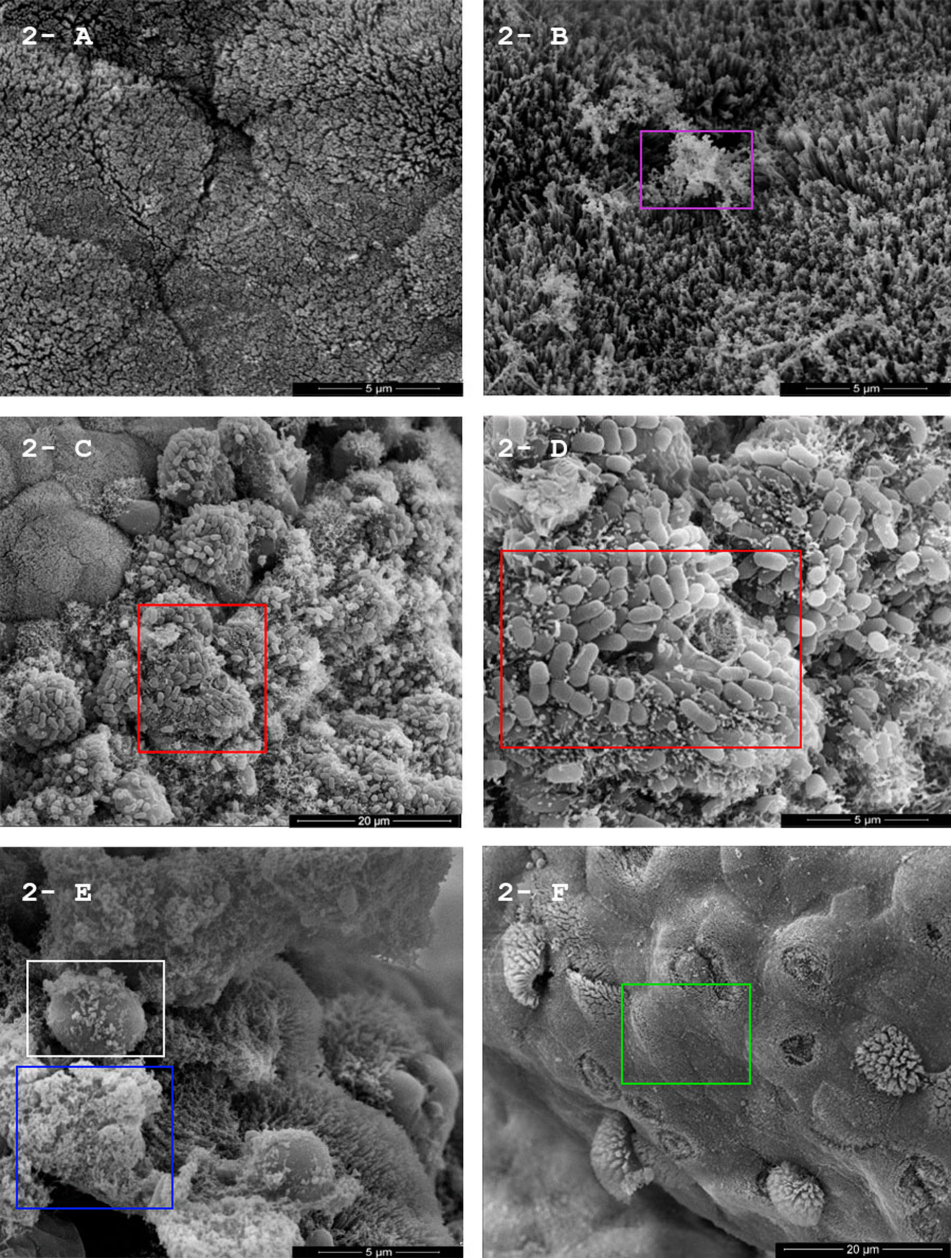
(A–F) Scanning Electron Microscopy (SEM) of small intestine in the inoculated rabbit ileal loops model. Micrograph of the intestinal rabbit mucosa 12 h post-*in vivo*-inoculation, observed by scanning electron microscopy. (A) Negative control (PBS) – Preserved intestinal epithelial structures with normal microvilli (15.000×). (B) Negative control (EC. DH5α). Preserved intestinal epithelial structures with normal microvilli (pink box) (20.000×). (C) clusters of adhering EC.2348/69 (positive control) (red box) (5.000×). (D) clusters of adhering EC.2348/69 (positive control) (red box) (15.000×). (E) macaw isolate MA-81- abundant mucous (blue box) and erythrocytes (white box) (15.000×). (F) macaw isolate MA-81 irregular epithelial intestinal surface showing loss of intestinal microvilli (green box) (5.000×).

The macroscopic changes observed in the rabbit ileal loops model showed that the macaw strain MA-81 induced fluid accumulation, distension, and hemorrhage with mucous and edema of intestine mucosa (+++) (Figure 1(A)). Positive control (EC2348/69) presented hyperemia and slight amount of mucus (+) (Figure 1(B)), and DH-5α and PBS respective negative controls showing no macroscopic alterations (Figure 1(C,D)). Scanning electron microscopy of the inoculated rabbit ileal loops demonstrated preservation of the intestinal epithelial structures in the negative control (Figure 2(A,B)), and many bacteria adhered in the positive control (EC2348/69) (Figure 2(C,D)). MA-81 inoculation demonstrated few adhered bacteria. However, a loss of intestinal villi is obvious (Figure 2(F)), the presence of erythrocytes, mucus, and scattered bacteria over an irregular epithelial intestinal surface (Figure 2(E)).

### 3.3. Histopathology

**Figure 3.**
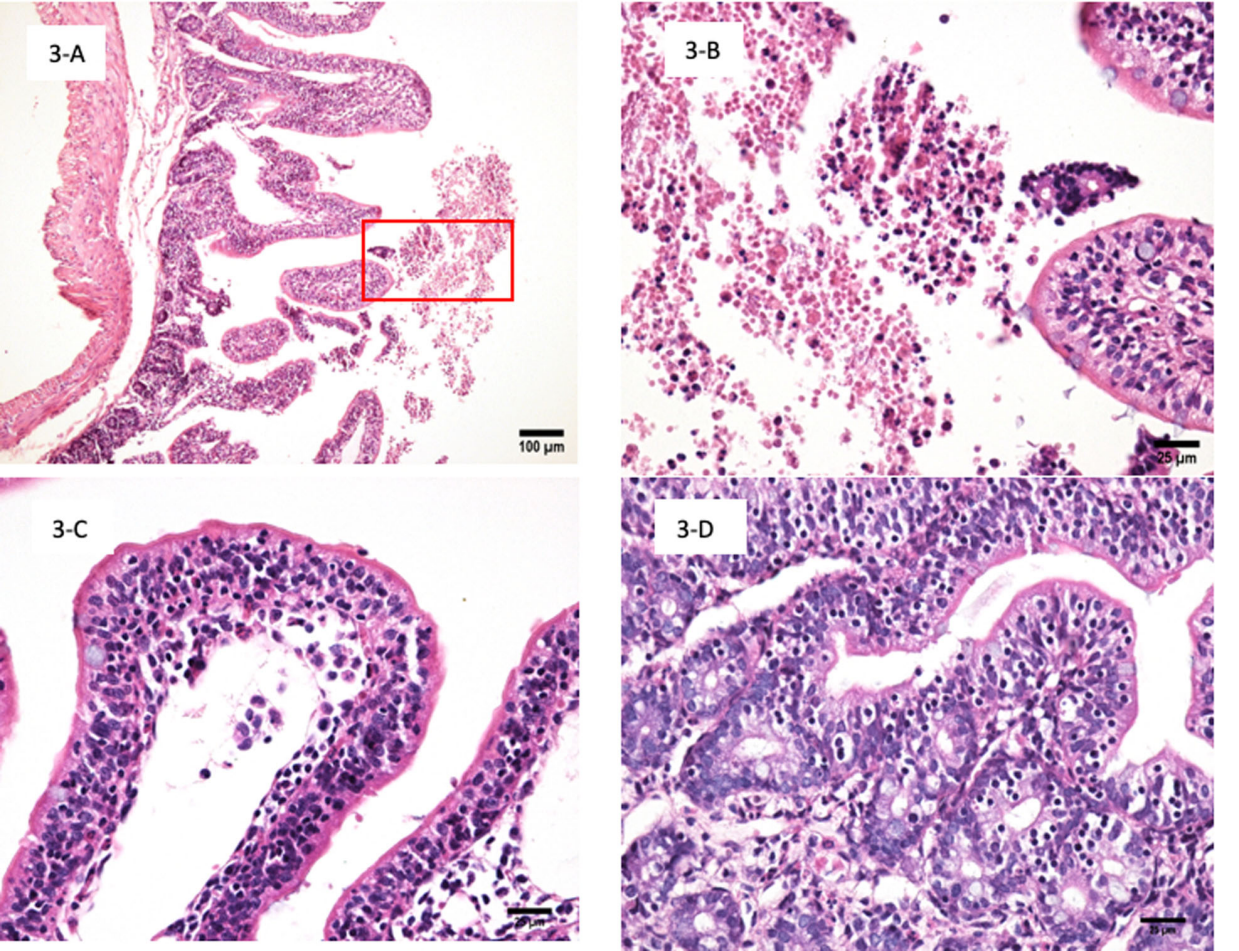
(A–D) Histopathology of the small intestine samples from inoculated rabbit ileal loop model with the MA-81 EPEC isolate, showing acute exudative enteritis. (A) Enteritis and partial reduction up to 20% in height of villus and with erythrocyte and heterophil exudate in the intestinal lumen (red box) 12 h after inoculation of macaw isolate MA-81 (HE, 100×). (B) Zoomed of red box mark of Figure A showing villus apex with heterophils, intra-epithelial lymphocytes, and mucus hemorrhagic exudate 12 h after inoculation of macaw isolate MA-81 (HE, 400×). (C) Detail of the villus tip presenting intra-epithelial lymphocytes and erythrocytes, lymphocytes, heterophils, and histiocytes 12 h after inoculation of macaw isolate MA-81 (HE, 400×). (D) Detail of small intestinal lamina propria, crypts and villus showing acute enteritis characterized by heterophils and lymphocytes in lamina propria and intra-epithelial lymphocytes in crypts and villus 12 h after inoculation of macaw isolate MA-81 (HE, 400×).

**Figure 4.**
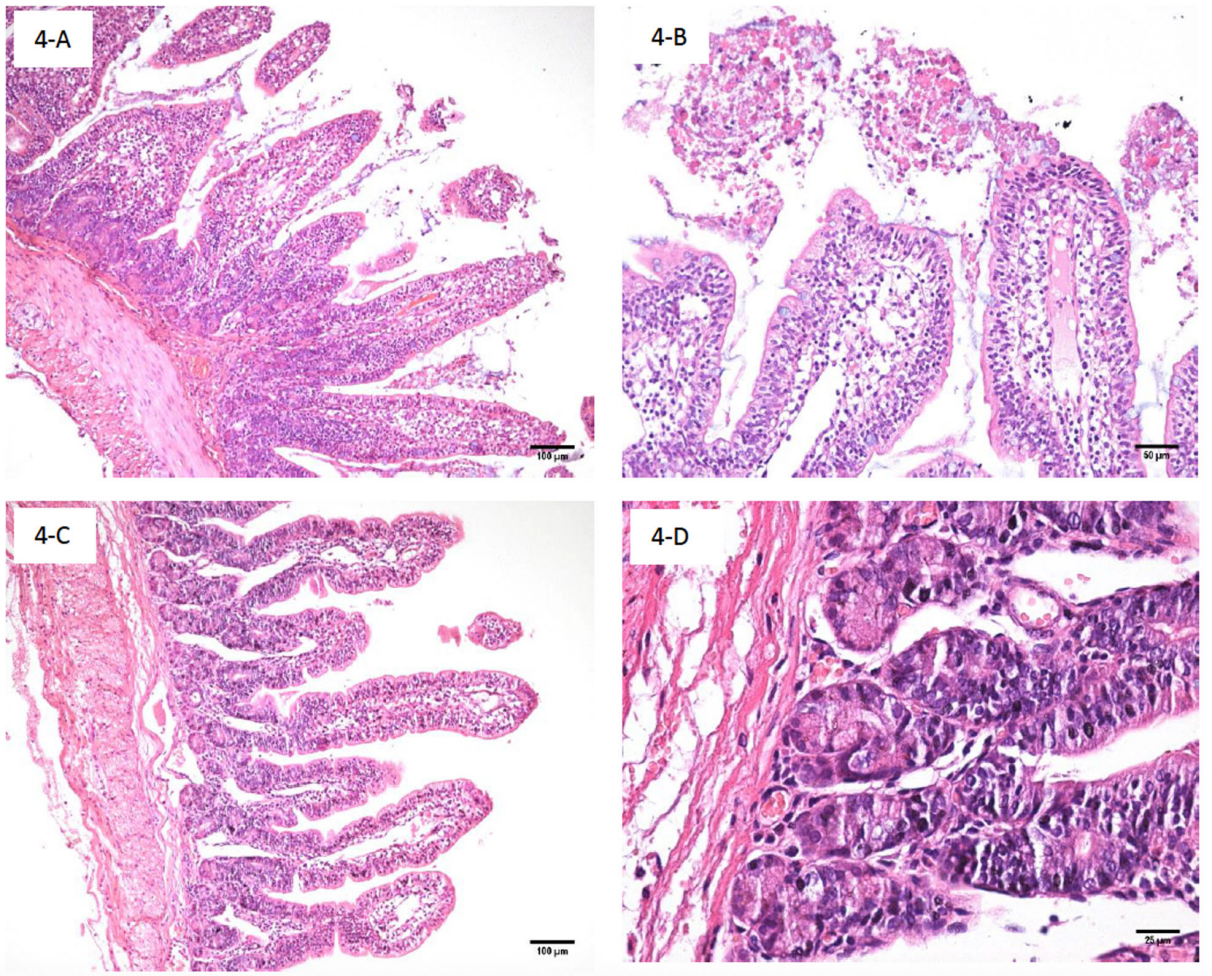
Histopathology of the small intestine samples from inoculated rabbit ileal loop model with positive (EC.2348/69) and negative (EC. K12 DH5α) control strains. (A) Small intestine sample of positive control (EC.2348/69) showing mucus, erythrocyte and leucocyte debris in lumen, HE, 100×; (B) Small intestine sample of positive control (EC.2348/69) at high magnification showing mucus, leucocyte and erythrocyte debris, HE, 200×; (C) Small intestine sample of negative control (DH5α). Note absence of lesion (HE, 100×); (D) Small intestine sample of negative control (DH5α) at crypt level lamina propria. Note the absence of lesions (HE, 400×).

Microscopic analysis of intestine inoculated with MA-81 showed moderate acute exudative and hemorrhagic enteritis characterized by intestinal villus height preserved for the most part of the intestinal segments with villi:crypt ratio of 5:1. However, multiple segments presented partial reduced villus height up to 20% (Figure 3(A–C)), and intestinal morphology is mildly altered by a marked number of intra-epithelial lymphocytes (IEL) in crypts and villus tips (up to 50 IEL/100 enterocytes) with heterophil exudate in inter crypts and villus lamina propria (Figure 3(C,D)). Increased number of heterophils, lymphocytes and histiocytes in the lamina propria of villus, marked multifocal hemorrhage with countless erythrocytes and heterophils mixed with mucous in the intestinal lumen (Figure 3(B,C)) were also observed without adhered bacteria in the brush border. Positive control (EC2348/69) presented foci of mild reduction of higher villi and absence of enteritis or changed number of IEL (up to 10 IEL/100 enterocytes) (Figure 4(A,B)). There was no bacteria adherence or inflammatory infiltrate in samples of negative controls (PBS or DH5α) (Figure 4(C,D)).

### 3.4. Phylogenomic comparisons

**Figure 5.**
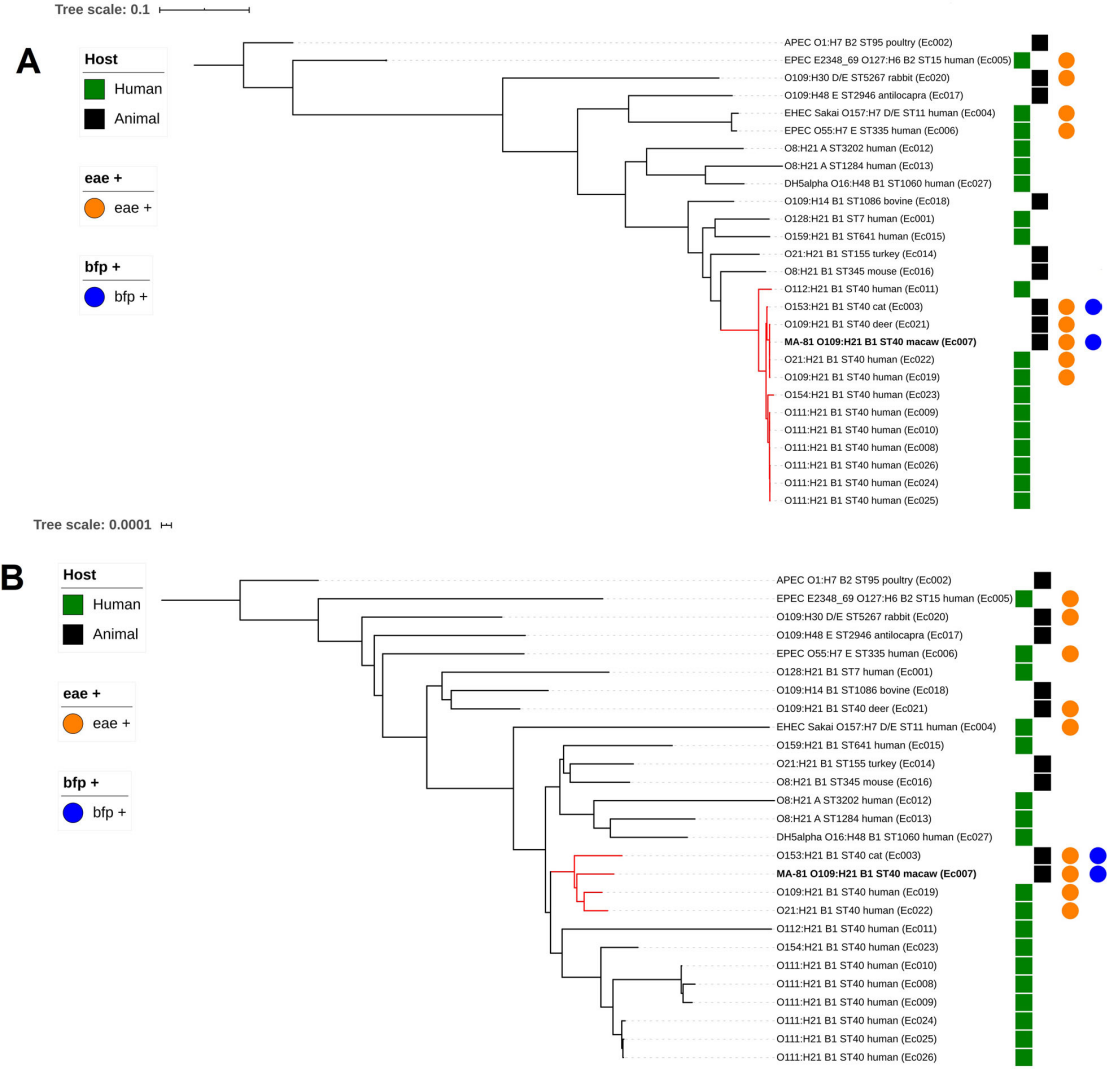
(A) Core genome phylogeny showing the correlation of the macaw isolate with human sequences and other animals in contact with anthropogenic activities. Phylogenetic tree constructed with the parSNP software and visualized on ITOL v.4 (https://itol.embl.de/). The sequences are classified according to host, the presence of the *eae* and *bfp* gene and classified according to different shapes and colors following the schematic diagrams. Clades containing the MA-81 isolate are highlighted in red with corresponding codes to each sequence of a given clade available in Table S1. (B) Whole genome phylogeny confirms the macaw isolate as highly related to two human clinical cases and a cat isolate. Phylogenetic tree constructed with the FFpry free alignment software and visualized on ITOL v.4 (https://itol.embl.de/). The sequences are classified according to host, the presence of the *eae* and *bfp* gene and classified according to different shapes and colors following the schematic diagrams. Clades containing the MA-81 isolate are highlighted in red with corresponding codes to each sequence of a given clade available in Table S1.

Comparison of the genome sequence of strain FMVZ-USP NL-81 with 26 *E. coli* genomes using core genome single nucleotide polymorphisms (SNPs) showed segregation of the different isolates primarily according to their phylogroup, while there was no particular association regarding hosts, with sequences from human and diverse animal species distributed throughout the tree (Figure 5(A)). The parrot sequence was found in a group of monophyletic clades that included phylogroup B1 sequences and somewhat related sequences having in common only the H21 flagellar antigen and, to a lesser extent, the O109 somatic antigen. The macaw sequence had a particularly strong association with clades belonging to the ST40 lineage located in a cluster of homologous O109:H21 ST40 EPEC isolates containing two human and one deer sequences and a related cat sequence.

For the whole genome alignment, there was again mostly an association of clades containing the H21 antigen, with diverse serotype combinations and host species, particularly in the subclades containing ST40 sequences (Figure 5(B)). Similarly, to the core genome SNP analysis, the same two human closely related sequences to the parrot strain and the cat sequence were found clustering together in the related subclades.

## 4. Discussion

This study reported a macaw as a carrier for typical EPEC, adding a first WGS characterization associated with the experimental mammal model, showing the severe intestinal effects of the inoculation of strain MA-81, isolated from pet bird. Different animal species have been recognized as zoonotic reservoirs of EPEC and a zoonotic transmission has been strongly suggested in the case of a diarrheic pet dog and an asymptomatic child sharing the same EPEC strain in a household (Rodrigues et al. 2004).

In the current study, the isolate was confirmed as a typical EPEC, as both the *eae* and *bfp*A genes were present (Pearson et al. 2016). Humans are considered the major reservoir for typical EPEC and this pathotype is still infrequently found in a wide variety of animal species (Schremmer et al. 1999; Moura et al. 2009; Sanches et al. 2017). Atypical strains are more commonly found in animals and are the most easily recognizable reservoirs for human infections (Krause et al. 2005).

In Brazil, typical and atypical strains have been recorded from healthy and sick captive and free-living birds (Knöbl et al. 2011; Saidenberg et al. 2012, 2017; Sanches et al. 2017).

Specific serotypes of EPEC strains have been reported as a cause of massive outbreaks affecting free-living songbirds involving the serotype O86 or captive reared partridges (serotype O103) (La Ragione et al. 2002, 2004). Our study shows that the macaw was colonized by *E. coli* O109:H21. This serotype of tEPEC and aEPEC has been described in clinical cases from humans and animals (Xu et al. 2017).

The O109 (and other O serogroups) of diverse H type strains are well known as a cause of EPEC diarrhea in rabbits (Blanco et al. 1996) and our experimental infection seem to show high tissue injuries in this animal model, although in a much more severe degree than the E2348/69 (Figures 1 and 2). These results suggested that the MA81 strain may be very pathogenic for mammals, but this hypothesis, until now, was supported only by the ileal loop experiment. Atypical EPEC belonging to a non-typeable O somatic antigen but H21 positive were also isolated from asymptomatic and symptomatic dogs in Brazil (Puño-Sarmiento et al. 2013) and a varied combination of the O109 or the H21 from animals considered potential reservoirs for human infections have also been published (Alonso et al. 2016, 2017).

The ST40 has been reported more commonly in human sources from clinical cases as shown by the sequences recovered from EnteroBase/NCBI databases (Supplementary Table 1), likely showing an over-representation of human sequences in our phylogenetic comparisons. Still, an EPEC ST40 cat isolate was also located in the nearby clade in both phylogenies and there was a homologous ST40 deer sequence in the SNP analysis. Xu et al. (2017) reported several atypical EPEC ST40 from asymptomatic humans and diarrhea cases in addition to urban birds’ fecal sources, suggesting zoonotic risks of this clonal lineage.

The MA-81 strain possesses virulence genes from the locus of enterocyte effacement pathogenicity island (LEE) (Supplementary Table 2) that codified the adhesin intimin (*eae*) and its translocated receptor Tir; the global regulator Ler; type three secretion system (T3SS): Esp-secreted proteins and non-Lee encoded factors (*cif, nle*). Other virulence determinants detected included the cytolethal distending toxin gene (*cdt*) whose function in EPEC virulence remains to be elucidated, among other genes encoding for additional adhesins and iron uptake systems (Supplementary Table 2).

Although the MA-81 strain had all the genetic components of a typical EPEC, which would cause classical A/E lesions, the extensive hemorrhage, inflammation, and mucus production prevented documentation using transmission electron microscopy. SEM illustrated few adhered bacteria, with free erythrocytes mixed with abundant inflammatory cells and mucus (Figure 2(E)). Copious mucus and hemorrhage were also observed in the intestinal contents (Figure 1(A)). In addition, histopathology revealed acute hemorrhagic enteritis with a marked increased number of IEL (Figure 3(A–D)). In contrast, infection with tEPEC E2348/69 presented many bacteria attached (Figure 2(C,D)) with less tissue injuries (Figure 1(B)) and inflammatory infiltrate in the lamina propria (Figure 4(A,B)). This report shows that MA-81 was quite pathogenic in the employed *in vivo* animal model.

It must be noted that the abundant hemorrhage and acute inflammatory lesion is not a characteristic of a typical EPEC, which is defined by secretory diarrhea with abundant mucus, loss of fluids and electrolytes (Torres 2017). Vieira et al. (2010, using the rabbit ligated ileal loops, reported that the strain 3991-1 (aEPEC) increased the production of mucins, and induced the attachment and effacement (A/E) lesion of the brush border 18 h after inoculation. The macroscopic findings are associated with hypersecretion of grayish fluid mucus, and the description of histological examination reported large clusters of bacteria in brush border. The authors do not mention hemorrhage or the presence of inflammatory cells in the lumen or lamina propria.

Sampaio et al. (2014) evaluated the ability of the aEPEC1711-4 to interact *in vivo* with rabbit after 8 and 24 h of exposition in ligated ileal loops, reporting A/E lesion, enterocyte invasion, and presence of intraluminal polymorphonuclear leukocytes. However, the authors did not mention the occurrence of hemorrhagic lesion. Gioia Di-Chiacchio et al. (2018) evaluated eight strains of *E. coli eae + bfp+* isolated from pet birds (*N. hollandicus* and *M. undulatus*). The macroscopic evaluation of rabbit intestine showed fluid accumulation and some strains induced the production of bloody mucus without inflammatory infiltrate in lamina propria. But these strains are considered hybrid of tEPEC and STEC, were positive for *stx*2f toxin and also presented cytotoxic effects on Vero cells, while EPEC MA-81 strain was negative for toxins, including Shiga-like toxins (STX) and cytotoxic necrotizing factor (CNF). In addition, the histopathology analysis showed a different pattern of MA-81, which was characterized by the ability to induce more intense hemorrhage and inflammatory process with IEL in villus tips.

The combined SEM and histopathologic results suggest the presence of other virulence mechanisms yet to be defined within this EPEC MA-81, at least in the *in vivo* experimental model employed here. Perhaps that might be related with putative new toxins and mechanisms of virulence, which we are currently unable to fully establish with our short-read sequencing results and *in vivo* experimental infection analysis. Effector proteins of the T3SS, particularly the Nle (present in this study’s isolate), are able to disrupt the cytoskeleton and tight junctions of enterocytes and also modulate or inhibit the pathways leading to the host’s inflammatory response (Gomes et al. 2016). We can postulate that the first effect may explain the hemorrhagic lesions and correlate to the histopathological findings. Regarding the inflammatory reaction, it remains to be elucidated its origin with more specific studies focusing on in-depth select or putative virulence determinants and expression/modulation of phenotypes.

Both phylogenies from the core and accessory genomes showed subclades with close relationships between the parrot strain and some human strains, in addition to a more distant relationship with the overall major clades including a few wild and domestic animal sequences (Figure 5(A,B)). The core genome analysis showed homology among the parrot and two humans and a deer ST40 atypical EPEC isolates as well as a close relation with a cat typical EPEC ST40 sequence (Figure 5(A)). Conversely, the whole genome analysis tended to a wider variety of serotypes and STs. Nevertheless, the related cat and the same two human sequences clustered together again in ST40 subclades close to the macaw sequence (Figure 5(B)). As there is a shortage of WGS EPEC sequences from birds to which we could compare our strain, further studies with strains from diverse species should be conducted to increase knowledge on the zoonotic potential.

## 5. Conclusions

As previously observed with other EPEC animal carriers, in particular, pets in close contact with humans, a zoonotic transmission is possible in certain circumstances (Rodrigues et al. 2004; Krause et al. 2005). Besides the genotypic similarities with EPEC that affect humans and animals and the observed phylogenetic correlations with sequences from humans and from animals in contact with anthropogenic activities, the isolate MA-81 tested here caused hemorrhagic enteritis in rabbit ileal loop model. This may suggest possible zoonotic risks for humans in contact with parrots that are carriers for such EPEC strains. These results demonstrate the need for improved hygienic husbandry methods for captive parrots, in order to reduce the risk of infection to humans.

## Supplementary Material

Supplemental Material

Supplemental Material
